# Maternal Risk Factors for Retinopathy of Prematurity in Korea: A Nationwide Population-Based Study

**DOI:** 10.1016/j.xops.2026.101088

**Published:** 2026-01-29

**Authors:** Hyeong Min Kim, Sang Hyun Park, Tae-Eun Kim, Hyun Jin Shin, Youn Hye Jo, Hyungwoo Lee

**Affiliations:** 1 Department of Ophthalmology, Konkuk University School of Medicine, Konkuk University Medical Center, Seoul, Republic of Korea; 2 Department of Clinical Pharmacology, Konkuk University Medical Center, Seoul, Republic of Korea

**Keywords:** Retinopathy of prematurity, Maternal, Risk factor, Nationwide, Cohort

## Abstract

**Purpose:**

To investigate the association between maternal health conditions and the risk of retinopathy of prematurity (ROP) in preterm infants using nationwide population-based data.

**Design:**

A retrospective cohort study.

**Participants:**

A total of 88 111 live preterm births in Korea between January 1, 2014, and December 31, 2017, identified from linked National Health Insurance Service and National Health Screening Program for Infants and Children databases.

**Methods:**

Multivariable logistic regression models were used to estimate adjusted odds ratios (aORs) for maternal risk factors associated with ROP and treatment-requiring ROP.

**Main Outcome Measures:**

Incidence of any ROP and treatment-requiring ROP.

**Results:**

Of 88 111 preterm infants, 17 181 (19.5%) developed ROP, and 340 (2.0% of ROP cases) required treatment. Maternal diabetes mellitus (aOR, 1.142; *P* < 0.001), dyslipidemia (aOR, 1.123; *P* < 0.001), hypertension (aOR, 1.092; *P* = 0.023), pelvic and genitourinary diseases (aOR, 1.172; *P* < 0.001), and delivery or placental disorders (aOR, 1.463; *P* < 0.001) were associated with higher odds of ROP. Among infants with ROP, ovarian dysfunction was associated with increased odds of treatment-requiring disease (aOR, 1.732; *P* < 0.001), whereas preeclampsia and gestational hypertension were associated with reduced odds (aOR, 0.465; *P* < 0.001).

**Conclusions:**

Multiple maternal conditions were associated with increased risk of any ROP in preterm infants. Among infants with ROP, ovarian dysfunction independently predicted progression to treatment-requiring disease, whereas preeclampsia and gestational hypertension were associated with reduced risk of severe ROP. Recognition of these maternal risk factors may support prenatal counseling, neonatal risk stratification, and targeted ROP screening.

**Financial Disclosure(s):**

The authors have no proprietary or commercial interest in any materials discussed in this article.

Retinopathy of prematurity (ROP) remains one of the leading causes of childhood blindness worldwide, affecting approximately 20 000 infants annually who become blind or severely visually impaired from this condition.^1^^,^^2^ Despite significant advances in neonatal care and screening protocols, the global burden of ROP continues to increase as survival rates of extremely premature infants improve, particularly in middle-income countries. Retinopathy of prematurity development reflects a multifactorial process involving maternal, prenatal, and neonatal influences that extend beyond the primary risk factors of prematurity and low birth weight.^3^, ^4^, ^5^

Current ROP screening guidelines primarily rely on gestational age and birth weight criteria, with most protocols recommending screening for infants born at ≤30 weeks gestational age or ≤1500 g birth weight.^3^ While neonatal risk factors for ROP have been extensively studied and consistently identified across populations—including respiratory distress syndrome, bronchopulmonary dysplasia, sepsis, and intraventricular hemorrhage—the role of maternal factors in ROP development has received less investigation.^6^, ^7^, ^8^, ^9^, ^10^, ^11^, ^12^ A few studies have examined maternal factors associated with ROP, with research indicating that maternal diabetes mellitus, hypertensive disorders of pregnancy (including gestational hypertension), and preeclampsia may be related to ROP development.^13^, ^14^, ^15^, ^16^, ^17^, ^18^ Nevertheless, extensive research has examined maternal risk factors associated with preterm delivery, with a recent nationwide population-based cohort study from Taiwan identifying maternal age, socioeconomic status, maternal allergic and autoimmune conditions, gynecological disorders, and pregnancy-related complications as significant predictors of preterm birth.^19^

This nationwide cohort study seeks to comprehensively examine maternal risk factors that contribute to ROP development and progression to treatment-requiring stages in preterm infants within South Korea. Understanding maternal contributions to ROP risk carries significant clinical implications: maternal health status may influence fetal development and growth, affecting retinal maturation in utero; these factors could enhance early risk assessment, improving screening efficiency and targeted interventions; and identifying modifiable maternal risk factors may guide clinical strategies in preconception and prenatal care to decrease ROP incidence.

## Methods

This study was approved by the Institutional Review Board of Konkuk University Medical Center (No. KUMC IRB 2025-07-021). The requirement for informed consent was waived by the institutional review board because this study used deidentified, nationwide administrative data with no direct patient contact or intervention. The study adhered to the principles outlined in the Declaration of Helsinki.

### Data Source and Study Cohort

This study utilized data from the National Health Insurance Service (NHIS) database and the National Health Screening Program for Infants and Children (NHSPIC) in South Korea. The NHIS database, covering over 97% of the South Korean population, provides comprehensive information on demographics, medical history, and medication records. Diagnoses are coded using the International Classification of Diseases, 10th revision (ICD-10). The NHSPIC is a nationwide surveillance program that includes medical history, physical examinations, anthropometric measurements, vision screening, and developmental assessment. All data were deidentified by the NHIS.

**Figure 1:**
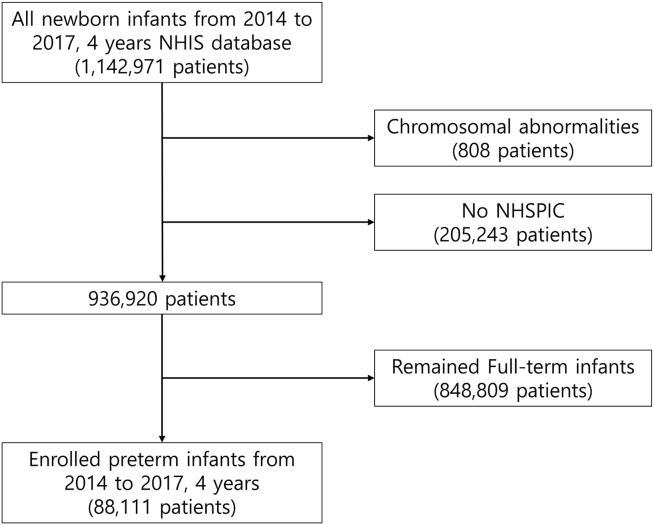
Flow chart of the enrolled study participants from the NHIS database and the NHSPIC in South Korea. NHIS = National Health Insurance Service; NHSPIC = National Health Screening Program for Infants and Children.

Mother–offspring dyads were obtained from the NHIS database, including all newborns and their mothers from January 1, 2014, to December 31, 2017. Among these, the preterm birth cohort identified using the NHSPIC was included in this study. Children diagnosed with chromosomal abnormalities (e.g., Down syndrome, Edwards syndrome, Patau syndrome, 5p deletion syndrome, Angelman syndrome, Klinefelter syndrome, and Turner syndrome) were excluded from this study (Fig 1).

### Clinical Variables

Retinopathy of prematurity cases were determined by ICD-10 code (H35.1), and the treatments of ROP were identified by procedure codes, laser photocoagulation (S5160 and S5161), cryotherapy (S5140), vitrectomy (S5121 and S5122), scleral buckling (S5130), and anti-VEGF intravitreal injection (S5070). Maternal comorbidities and obstetric complications were defined using ICD-10 codes as follows: tuberculosis (A15–A19), sexually transmitted diseases (A50–A64), viral infections excluding hepatitis and human immunodeficiency virus (B00–B09), hepatitis (B15–B19), human immunodeficiency virus (B20–B24), uterine myoma (D25), anemia (D50–D64), thyroid disorders (E00–E07), diabetes mellitus (E10–E14), ovarian dysfunction (E28), obesity and hyperalimentation (E66), dyslipidemia (E78), hypertension (I10–I15), coronary artery diseases (I20–I25), cerebrovascular diseases (I60–I69), heart diseases (I50–I59), rheumatic diseases (M05, M32, M35, M45), pelvic and genitourinary disorders (N70–N99), pregnancy-related complications (O10–O48), proteinuria and gestational hypertension, including preeclampsia (O10–O16), other specified complications of pregnancy (O20–O29), and delivery or placental disorders (O30-O48). Neonatal adverse outcomes were also defined using ICD-10 codes, including premature rupture of membranes (P01.1), chorioamnionitis (P02.7), respiratory distress syndrome (P22), apnea of prematurity (P28.4), chronic lung disease/bronchopulmonary dysplasia (P27), anemia of prematurity (P61), intraventricular hemorrhage (P52.0–P52.3), bacterial sepsis of newborn (P36), necrotizing enterocolitis (P77), and neonatal thrombocytopenia (P61.0–P61.1). Birth weights were retrieved from the NHSPIC.

### Statistical Analysis

The comparison of baseline characteristics between non-ROP and ROP groups and between observed and treated ROP groups were performed using *t*-test for continuous variables and chi-squared test for categorical variables. Odds ratios with 95% confidence intervals (CIs) were estimated using multivariate logistic regression model. The models were further adjusted for maternal age, neonatal gender, mode of delivery, multiparity, birth weight, and maternal diseases. Maternal age was categorized as <35 years or ≥35 years. Birth weight was entered as a categorical variable in the following groups: <1000 g, 1000 to 1499 g, 1500 to 1999 g, 2000 to 2499 g, and ≥2500 g. Gestational age data were not available in the NHSPIC database structure. Because birth weight and gestational age are highly correlated, simultaneous inclusion of both variables in the multivariable model would introduce problematic multicollinearity. Therefore, birth weight—a well-validated surrogate marker for prematurity severity—was employed as the primary neonatal adjustment variable. All statistical analyses were performed using SAS 9.4 (SAS Institute Inc.).

## Results

### Clinical Characteristics of Prematurity Groups

Between January 1, 2014, and December 31, 2017, a total of 88 111 preterm births were evaluated for ROP. Among these, 70 930 infants did not develop ROP, while 17 181 (19.5%) were diagnosed with ROP. Of the cases diagnosed with ROP, 340 (1.98%) received treatment. Compared with preterm infants without ROP, those with ROP differed in maternal age, neonatal sex, delivery mode, multiparity, and birth weight (Table 1). In comparisons of observed versus treatment-requiring ROP, birth weight emerged as the sole variable showing a significant difference. Specifically, the proportions of extremely low birth weight (<1000 g; 2.90% vs 43.8%) and very low birth weight (1000–1499 g; 16.3% vs 34.4%) were distinctly higher in the treated ROP group. These findings reveal that the treated ROP reflects more severe cases of preterm birth, which are closely associated with lower birth weights.

**Table 1:** Demographics and Clinical Characteristics of Study Subjects

	All (N = 88 111)	Non-ROP (n = 70 930)	ROP (n= 17 181)	*P* Value	Observed ROP (n = 16 841)	Treated ROP (n = 340)	*P* Value
Maternal age, yrs	33.24 ± 4.13	33.18 ± 4.12	33.48 ± 4.15	**<0.001**	33.48 ± 4.15	33.84 ± 4.16	0.106
<35 yrs	53 625 (60.9%)	43 652 (61.5%)	9973 (58.1%)	**<0.001**	9786 (58.1%)	187 (55.0%)	0.250
≥35 yrs	34 486 (39.1%)	27 278 (38.5%)	7208 (41.9%)		7055 (41.9%)	153 (45.0%)	
Neonatal gender, male (%)	46 491 (52.8%)	36 991 (52.2%)	9500 (55.3%)	**<0.001**	9319 (55.3%)	181 (53.2%)	0.441
Mode of delivery cesarean section (%)	62 345 (70.8%)	49 041 (69.1%)	13 304 (77.4%)	**<0.001**	13 052 (77.5%)	252 (74.1%)	0.139
Pregnancy, multiparity (%)	32 595 (36.9%)	26 122 (36.8%)	6473 (37.7%)	**0.039**	6359 (37.8%)	114 (33.5%)	0.111
Birth weight, N (%), g							
<1000	774 (0.88%)	142 (0.20%)	632 (3.68%)	**<0.001**	483 (2.90%)	149 (43.8%)	**<0.001**
1000–1499	4189 (4.75%)	1326 (1.87%)	2863 (16.7%)		2746 (16.3%)	117 (34.4%)	
1500–1999	14 014 (15.9%)	7929 (11.2%)	6085 (35.4%)		6033 (35.8%)	52 (15.3%)	
2000–2499	35 420 (40.2%)	30 007 (42.3%)	5413 (31.5%)		5402 (32.1%)	11 (3.20%)	
≥2500	33 714 (38.3%)	31 526 (44.5%)	2188 (12.7%)		2177 (12.9%)	11 (3.20%)	

ROP = retinopathy of prematurity.

*P* values in boldface indicate statistical significance.

### Maternal and Neonatal Risk Factors Associated with ROP

Univariate analysis was conducted to evaluate maternal and neonatal risk factors associated with ROP (Table 2). Twenty-one maternal comorbidities and 10 neonatal conditions were considered. In comparison to the non-ROP group, a higher prevalence of specific maternal factors was observed in the ROP group, including uterine myoma (8.62% vs 9.86%, *P* < 0.001), anemia (31.8% vs 33.5%, *P* < 0.001), thyroid diseases (38.1% vs 40.4%, *P* < 0.001), diabetes mellitus (9.04% vs 10.9%, *P* < 0.001), dyslipidemia (18.8% vs 21.3%, *P* < 0.001), hypertension (5.70% vs 8.50%, *P* < 0.001), coronary artery diseases (1.47% vs 1.71%, *P* = 0.025), rheumatic diseases (1.36% vs 1.76%, *P* < 0.001), pelvic and genitourinary diseases (89.2% vs 90.8%, *P* < 0.001), pregnancy-related complications including preeclampsia and gestational hypertension (24.8% vs 28.5%, *P* < 0.001), other maternal disorders (82.0% vs 82.7%, *P* = 0.035), and delivery or placental disorders (88.5% vs 93.5%, *P* < 0.001). Notably, when comparing preterm births with observed versus treated ROP, ovarian dysfunction (12.0% vs 18.5%, *P* < 0.001) was significantly more common among those who required treatment, whereas preeclampsia and gestational hypertension (28.6% vs 20.6%, *P* < 0.001) were less common.

**Table 2:** Univariate Analysis of Risk Factors Associated with ROP

	All (N = 88 111)	Non-ROP (n = 70 930)	ROP (n = 17 181)	*P* Value	Observed ROP (n = 16 841)	Treated ROP (n = 340)	*P* Value
Maternal risk factors							
Tuberculosis	339 (0.38%)	283 (0.40%)	56 (0.33%)	0.165	55 (0.30%)	1 (0.30%)	0.917
Sexually transmitted diseases	20 042 (22.8%)	16 038 (22.6%)	4004 (23.3%)	0.051	3929 (23.3%)	75 (22.1%)	0.583
Viral infection	7666 (8.70%)	6204 (8.75%)	1462 (8.51%)	0.322	1437 (8.50%)	25 (7.40%)	0.440
Hepatitis	6553 (7.44%)	5278 (7.44%)	1275 (7.42%)	0.928	1249 (7.40%)	26 (7.60%)	0.872
HIV	139 (0.16%)	111 (0.16%)	28 (0.16%)	0.848	28 (0.20%)	0 (0.00%)	0.452
Uterine myoma	7806 (8.86%)	6112 (8.62%)	1694 (9.86%)	**<0.001**	1653 (9.80%)	41 (12.1%)	0.169
Anemia	28 280 (32.1%)	22 534 (31.8%)	5746 (33.5%)	**<0.001**	5637 (33.5%)	109 (32.1%)	0.584
Thyroid diseases	33 935 (38.5%)	26 993 (38.1%)	6942 (40.4%)	**<0.001**	6802 (40.4%)	140 (41.2%)	0.769
Diabetes mellitus	8288 (9.41%)	6413 (9.04%)	1875 (10.9%)	**<0.001**	1833 (10.9%)	42 (12.4%)	0.389
Ovarian dysfunction	10 670 (12.1%)	8591 (12.1%)	2079 (12.1%)	0.967	2016 (12.0%)	63 (18.5%)	**<0.001**
Obesity and hyperalimentation	461 (0.52%)	358 (0.50%)	103 (0.60%)	0.122	100 (0.60%)	3 (0.90%)	0.495
Dyslipidemia	17 001 (19.3%)	13 340 (18.8%)	3661 (21.3%)	**<0.001**	3602 (21.4%)	59 (17.4%)	0.072
Hypertension	5502 (6.24%)	4041 (5.70%)	1461 (8.50%)	**<0.001**	1437 (8.50%)	24 (7.10%)	0.334
Coronary artery diseases	1338 (1.52%)	1045 (1.47%)	293 (1.71%)	**0.025**	287 (1.70%)	6 (1.80%)	0.932
Cerebrovascular diseases	613 (0.70%)	486 (0.69%)	127 (0.74%)	0.444	124 (0.70%)	3 (0.90%)	0.755
Heart diseases	5024 (5.70%)	4053 (5.71%)	971 (5.65%)	0.751	950 (5.60%)	21 (6.20%)	0.672
Rheumatic diseases	1270 (1.44%)	967 (1.36%)	303 (1.76%)	**<0.001**	297 (1.80%)	6 (1.80%)	0.998
Pelvic and genitourinary diseases	78 852 (89.5%)	63 260 (89.2%)	15 592 (90.8%)	**<0.001**	15 276 (90.7%)	316 (92.9%)	0.159
Pregnancy-related complications							
Preeclampsia and hypertension	22 464 (25.5%)	17 576 (24.8%)	4888 (28.5%)	**<0.001**	4818 (28.6%)	70 (20.6%)	**0.001**
Other maternal disorders	72 379 (82.2%)	58 171 (82.0%)	14 208 (82.7%)	**0.035**	13 929 (82.7%)	279 (82.1%)	0.753
Delivery or placental disorders	78 853 (89.5%)	62 797 (88.5%)	16 056 (93.5%)	**<0.001**	15 740 (93.5%)	316 (92.9%)	0.701
Neonatal risk factors							
Premature rupture of membranes	5556 (6.31%)	3535 (4.98%)	2021 (11.8%)	**<0.001**	1977 (11.7%)	44 (12.9%)	0.495
Chorioamnionitis	815 (0.92%)	336 (0.47%)	479 (2.79%)	**<0.001**	461 (2.70%)	18 (5.30%)	**0.004**
Respiratory distress syndrome	12 335 (14.0%)	4887 (6.89%)	7448 (43.4%)	**<0.001**	7163 (42.5%)	285 (83.8%)	**<0.001**
Apnea	7278 (8.26%)	2822 (3.98%)	4456 (25.9%)	**<0.001**	4280 (25.4%)	176 (51.8%)	**<0.001**
Chronic respiratory disease/ bronchopulmonary dysplasia	2841 (3.22%)	928 (1.31%)	1913 (11.1%)	**<0.001**	1732 (10.3%)	181 (53.2%)	**<0.001**
Anemia	5639 (6.40%)	2405 (3.39%)	3234 (18.8%)	**<0.001**	3114 (18.5%)	120 (35.3%)	**<0.001**
Intraventricular hemorrhage	3742 (4.25%)	1783 (2.51%)	1959 (11.4%)	**<0.001**	1866 (11.1%)	93 (27.4%)	**<0.001**
Bacterial sepsis of newborn	8672 (9.84%)	4413 (6.22%)	4259 (24.8%)	**<0.001**	4118 (24.5%)	141 (41.5%)	**<0.001**
Necrotizing enterocolitis	874 (0.99%)	318 (0.45%)	556 (3.24%)	**<0.001**	510 (3.00%)	46 (13.5%)	**<0.001**
Thrombocytopenia	591 (0.67%)	307 (0.43%)	284 (1.65%)	**<0.001**	261 (1.50%)	23 (6.80%)	**<0.001**

HIV = human immunodeficiency virus; ROP = retinopathy of prematurity.

*P* values in boldface indicate statistical significance.

For neonatal risk factors, all 10 comorbidities showed a significantly higher prevalence in the ROP groups compared to non-ROP preterm births (all *P* < 0.001). Furthermore, when stratified between observed and treated ROP, 9 out of 10 neonatal comorbidities—except premature rupture of membranes—remained significantly different. This trend indicates that, similar to prior studies, the majority of neonatal comorbidities increase according to the severity of preterm status: highest in treated ROP, followed by observed ROP, and lowest in non-ROP preterm births.

### Multivariate Analysis of Maternal Risk Factors Associated with ROP

Given that neonatal risk factors for preterm birth and ROP are well-established in previous studies, this investigation prioritized identifying maternal contributors. Multivariate logistic regression analysis was conducted (Table 3), with adjusted odds ratios (aORs) for maternal age, neonatal gender, delivery method, multiparity, birth weight, and 21 maternal comorbidities. Results showed that, compared to the non-ROP group, several maternal conditions were significantly associated with increased risk for ROP: diabetes mellitus (aOR 1.142, 95% CI 1.068–1.221, *P* < 0.001), dyslipidemia (aOR 1.123, 95% CI 1.067–1.182, *P* < 0.001), hypertension (aOR 1.092, 95% CI 1.012–1.178, *P* = 0.023), pelvic and genitourinary diseases (aOR 1.172, 95% CI 1.097–1.251, *P* < 0.001), and delivery or placental disorders (aOR 1.463, 95% CI 1.359–1.576, *P* < 0.001). In the analysis comparing observed ROP versus treatment-requiring ROP, ovarian dysfunction was significantly associated with increased odds of progression to severe ROP requiring treatment (aOR 1.732, 95% CI 1.262–2.378, *P* < 0.001). Conversely, among infants with established ROP, preeclampsia and gestational hypertension were associated with reduced odds of progressing to treatment-requiring disease (aOR 0.465, 95% CI 0.341–0.634, *P* < 0.001).

**Table 3:** Multivariate-Adjusted OR of Maternal Risk Factors Associated with ROP

Maternal risk factors	Non-ROP vs ROP			Observed ROP vs Treated ROP		
	Adjusted OR	95% CI	*P* Value	Adjusted OR	95% CI	*P* Value
Tuberculosis	0.816	0.613–1.088	0.166	1.032	0.123–8.627	0.976
Sexually transmitted diseases	1.038	0.988–1.090	0.135	0.899	0.679–1.191	0.457
Viral infection	0.995	0.930–1.064	0.872	0.858	0.553–1.332	0.494
Hepatitis	0.997	0.936–1.063	0.928	1.051	0.676–1.633	0.824
HIV	1.275	0.809–2.008	0.294	-	-	-
Uterine myoma	1.013	0.950–1.081	0.692	1.133	0.789–1.626	0.499
Anemia	1.011	0.971–1.053	0.591	0.925	0.719–1.189	0.542
Thyroid diseases	1.010	0.970–1.052	0.617	0.987	0.772–1.262	0.915
Diabetes mellitus	1.142	1.068–1.221	**<0.001**	1.295	0.880–1.905	0.189
Ovarian dysfunction	0.999	0.949–1.051	0.967	1.732	1.262–2.378	**<0.001**
Obesity and hyperalimentation	1.189	0.954–1.481	0.122	1.703	0.495–5.856	0.398
Dyslipidemia	1.123	1.067–1.182	**<0.001**	0.802	0.571–1.126	0.202
Hypertension	1.092	1.012–1.178	**0.023**	0.778	0.483–1.254	0.302
Coronary artery diseases	0.964	0.828–1.123	0.637	1.023	0.413–2.532	0.961
Cerebrovascular diseases	0.903	0.722–1.131	0.374	1.104	0.315–3.872	0.877
Heart diseases	0.993	0.916–1.077	0.865	1.339	0.827–2.167	0.235
Rheumatic diseases	1.022	0.880–1.187	0.776	0.821	0.339–1.987	0.662
Pelvic and genitourinary diseases	1.172	1.097–1.251	**<0.001**	1.214	0.773–1.906	0.399
Pregnancy-related complications						
Preeclampsia and hypertension	1.025	0.977–1.075	0.319	0.465	0.341–0.634	**<0.001**
Other maternal disorders	1.048	0.997–1.102	0.067	1.145	0.843–1.555	0.386
Delivery or placental disorders	1.463	1.359–1.576	**<0.001**	0.666	0.417–1.065	0.089

CI = confidence interval; HIV = human immunodeficiency virus; OR = odds ratio; ROP = retinopathy of prematurity.

*P* values in boldface indicate statistical significance.

## Discussion

This nationwide, population-based analysis of 88 111 preterm deliveries in South Korea over 4 years provides the most comprehensive evaluation to date of maternal contributors to ROP. We identified multiple maternal comorbidities—diabetes mellitus, dyslipidemia, hypertension, pelvic and genitourinary diseases, and delivery or placental disorders—were associated with higher odds of ROP. Importantly, ovarian dysfunction stood out as an independent risk factor for progression to treatment-requiring ROP. In contrast, preeclampsia and gestational hypertension were associated with a potentially protective effect on severe ROP.

Chronic maternal conditions—including diabetes mellitus, dyslipidemia, and hypertension—were linked to increased odds of ROP, suggesting that maternal metabolic dysfunction may influence fetal retinal vascular development. Moreover, pregnancies complicated by these chronic disorders are more likely to result in preterm birth and fetal growth restriction—both independent risk factors for ROP—and are characterized by a proinflammatory intrauterine environment that may further disrupt retinal angiogenesis through excess cytokine activity.^20^^,^^21^ Likewise, pelvic and genitourinary disorders in pregnancy—such as urinary tract infections, pelvic inflammatory disease, or cervical insufficiency—may also increase ROP risk primarily by promoting intrauterine inflammation and precipitating preterm birth. Inflammatory cytokines released during maternal infection can cross the placenta, triggering a fetal inflammatory response that disrupts normal retinal angiogenesis. Concurrently, many of these conditions lead to cervical changes or chorioamnionitis, increasing the likelihood of early delivery and fetal growth restriction—both well-established ROP risk factors.^22^

Studies examining hypertensive disorders of pregnancy as ROP risk factors have produced inconsistent findings.^17^^,^^23^^,^^24^ Seiberth and Linderkamp observed that maternal preeclampsia, along with antenatal betamethasone–induced lung maturation, was linked to a lower ROP incidence, proposing that maternal stress and elevated cortisol accelerate maturation in very-low-birth-weight infants.^24^ Fortes Filho et al similarly reported that preeclampsia conferred protection against severe ROP, hypothesizing that the antiangiogenic state and reduced VEGF levels in preeclampsia inhibit aberrant retinal neovascularization.^14^ In contrast, a recent meta-analysis covering 13 cohort studies and 45 000 infants found no overall association between hypertensive pregnancy disorders and ROP.^25^ In our cohort, importantly, preeclampsia and gestational hypertension did not significantly influence the overall development of ROP (aOR 1.025, *P* = 0.319)—findings that align with the meta-analysis. However, in the stratified analysis of infants who already developed ROP, these conditions were associated with substantially reduced odds of progressing to treatment-requiring severity (aOR 0.465, *P* < 0.001), which is consistent with Fortes Filho et al's hypothesis regarding the antiangiogenic protective effect in severe disease.

Our identification of ovarian dysfunction as the sole maternal risk factor for treatment-requiring ROP represents a novel finding with potentially important clinical implications. Ovarian dysfunction presents a range of conditions affecting ovarian hormone production and function, including polycystic ovary syndrome, premature ovarian insufficiency, and other endocrine disorders. The mechanism by which ovarian dysfunction might influence severe ROP development is not immediately apparent but could involve several pathways. Ovarian dysfunction is associated with altered steroid hormone production, which could affect fetal development and maternal–fetal nutrient exchange. Additionally, conditions causing ovarian dysfunction often involve systemic inflammation and metabolic disruption, which may contribute to adverse pregnancy outcomes, including preterm birth and fetal growth restriction.^26^ The specific association with treatment-requiring ROP suggests that ovarian dysfunction may influence the severity of retinal vascular pathology rather than simply increasing the overall risk of ROP development.

This study's major strengths include its nationwide population-based design, large sample size, and comprehensive assessment of maternal risk factors using standardized diagnostic codes. The use of the NHIS database ensures virtually complete case ascertainment and minimizes selection bias. The ability to distinguish between any ROP and treatment-requiring ROP provides important insights into factors associated with disease severity. However, this study has several important limitations. Retinopathy of prematurity was defined by ICD-10 code H35.1, which may capture screened infants without active disease, potentially overestimating incidence—the unexpectedly high ROP proportion in infants with birth weight ≥2000 g supports this concern. Gestational age data were unavailable, so birth weight served as a categorical surrogate for prematurity severity; including both would create multicollinearity and may introduce residual confounding. Anti-VEGF treatment relied on procedure code S5070, and off-label bevacizumab use during 2014–2017 may not have been consistently coded, potentially underdetecting treatment-requiring cases. As an administrative database study, we lacked detailed clinical information including ROP severity classification, disease onset timing, maternal glycemic control, and specific treatment details. The diagnostic accuracy of ICD-10 codes for maternal conditions likely varied across institutions, and we could not account for unmeasured confounders such as socioeconomic status, maternal nutrition, or environmental exposures. Finally, mother–infant linkage and NHSPIC participation further restricted the cohort, potentially limiting generalizability.

In summary, this nationwide cohort study identified maternal diabetes mellitus, dyslipidemia, hypertension, pelvic and genitourinary diseases, and pregnancy-related complications as maternal risk factors for ROP in preterm infants. Notably, ovarian dysfunction independently predicted progression to treatment-requiring ROP, highlighting the role of maternal endocrine status in severe disease pathogenesis. These observations may inform prenatal counseling and support risk stratification and targeted neonatal ophthalmic surveillance among at-risk preterm infants.

## References

[1] Bhatnagar A., Skrehot H.C., Bhatt A. (2023). Epidemiology of retinopathy of prematurity in the US from 2003 to 2019. JAMA Ophthalmol.

[2] Na K.H., Kim K.H., Kang T.U. (2020). Incidence, long-term visual outcomes, and mortality in retinopathy of prematurity in Korea: a nationwide population-based study. Invest Ophthalmol Vis Sci.

[3] Fierson W.M., American Academy of Pediatrics Section on Ophthalmology, American Academy of Ophthalmology, American Association for Pediatric Ophthalmology and Strabismus, American Association of Certified Orthoptists (2018). Screening examination of premature infants for retinopathy of prematurity. Pediatrics.

[4] Kim S.J., Port A.D., Swan R. (2018). Retinopathy of prematurity: a review of risk factors and their clinical significance. Surv Ophthalmol.

[5] van Sorge A.J., Schalij-Delfos N.E., Kerkhoff F.T. (2013). Reduction in screening for retinopathy of prematurity through risk factor adjusted inclusion criteria. Br J Ophthalmol.

[6] Akkoyun I., Oto S., Yilmaz G. (2006). Risk factors in the development of mild and severe retinopathy of prematurity. J AAPOS.

[7] Freitas A.M., Morschbacher R., Thorell M.R., Rhoden E.L. (2018). Incidence and risk factors for retinopathy of prematurity: a retrospective cohort study. Int J Retina Vitreous.

[8] Holmstrom G., Broberger U., Thomassen P. (1998). Neonatal risk factors for retinopathy of prematurity--a population-based study. Acta Ophthalmol Scand.

[9] Holmstrom G., Tornqvist K., Al-Hawasi A. (2018). Increased frequency of retinopathy of prematurity over the last decade and significant regional differences. Acta Ophthalmol.

[10] Kang E.Y., Lien R., Wang N.K. (2018). Retinopathy of prematurity trends in Taiwan: a 10-year nationwide population study. Invest Ophthalmol Vis Sci.

[11] Quinn M.K., Lee H.C., Profit J., Chu A. (2024). Trends in retinopathy of prematurity among preterm infants in California, 2012 to 2021. JAMA Ophthalmol.

[12] van Sorge A.J., Termote J.U., Kerkhoff F.T. (2014). Nationwide inventory of risk factors for retinopathy of prematurity in the Netherlands. J Pediatr.

[13] Bental Y., Reichman B., Shiff Y. (2011). Impact of maternal diabetes mellitus on mortality and morbidity of preterm infants (24-33 weeks' gestation). Pediatrics.

[14] Fortes Filho J.B., Costa M.C., Eckert G.U. (2011). Maternal preeclampsia protects preterm infants against severe retinopathy of prematurity. J Pediatr.

[15] Gagliardi L., Rusconi F., Bellu R. (2014). Association of maternal hypertension and chorioamnionitis with preterm outcomes. Pediatrics.

[16] Holmstrom G., Thomassen P., Broberger U. (1996). Maternal risk factors for retinopathy of prematurity--a population-based study. Acta Obstet Gynecol Scand.

[17] Ozkan H., Cetinkaya M., Koksal N. (2011). Maternal preeclampsia is associated with an increased risk of retinopathy of prematurity. J Perinat Med.

[18] Tunay Z.O., Ozdemir O., Acar D.E. (2016). Maternal diabetes as an independent risk factor for retinopathy of prematurity in infants with birth weight of 1500 g or more. Am J Ophthalmol.

[19] Wu S.T., Lin C.H., Lin Y.H. (2024). Maternal risk factors for preterm birth in Taiwan, a nationwide population-based cohort study. Pediatr Neonatol.

[20] Robert M.F., Neff R.K., Hubbell J.P. (1976). Association between maternal diabetes and the respiratory-distress syndrome in the newborn. N Engl J Med.

[21] Schrufer T.L., Antonetti D.A., Sonenberg N. (2010). Ablation of 4E-BP1/2 prevents hyperglycemia-mediated induction of VEGF expression in the rodent retina and in muller cells in culture. Diabetes.

[22] Huang C.C., Huang C.C., Lin S.Y. (2019). Association of pelvic inflammatory disease (PID) with ectopic pregnancy and preterm labor in Taiwan: a nationwide population-based retrospective cohort study. PLoS One.

[23] Gotsch F., Romero R., Kusanovic J.P. (2008). Preeclampsia and small-for-gestational age are associated with decreased concentrations of a factor involved in angiogenesis: soluble Tie-2. J Matern Fetal Neonatal Med.

[24] Seiberth V., Linderkamp O. (2000). Risk factors in retinopathy of prematurity. a multivariate statistical analysis. Ophthalmologica.

[25] Zhu T., Zhang L., Zhao F. (2017). Association of maternal hypertensive disorders with retinopathy of prematurity: a systematic review and meta-analysis. PLoS One.

[26] Saadati S., Mason T., Godini R. (2025). Metformin use in women with polycystic ovary syndrome (PCOS): opportunities, benefits, and clinical challenges. Diabetes Obes Metab.

